# Improved detection of biomarkers in cervico-vaginal mucus (CVM) from postpartum cattle

**DOI:** 10.1186/s12917-018-1619-5

**Published:** 2018-09-29

**Authors:** Mounir Adnane, Paul Kelly, Aspinas Chapwanya, Kieran G. Meade, Cliona O’Farrelly

**Affiliations:** 1 Comparative Immunology Group, School of Biochemistry and Immunology, Trinity College, Dublin, Ireland; 2 Institute of Veterinary Sciences, Tiaret, Algeria; 3 Department of Clinical Sciences, Ross University School of Veterinary Medicine, Basseterre, West Indies St. Kitts and Nevis; 4 Animal & Bioscience Research Department, Animal & Grassland Research and Innovation Centre, Teagasc, Grange, Co. Meath, Ireland; 5 Immunogenetics & Animal Health, Animal & Grassland Research and Innovation Centre, Teagasc, Grange, Co. Meath, Ireland

**Keywords:** Cervico-vaginal mucus, Postpartum, Reducing agent, Endometritis, Biomarker

## Abstract

**Background:**

In the postpartum cow, early diagnosis of uterine disease is currently problematic due to the lack of reliable, non-invasive diagnostic methods. Cervico-vaginal mucus (CVM) is an easy to collect potentially informative source of biomarkers for the diagnosis and prognosis of uterine disease in cows. Here, we report an improved method for processing CVM from postpartum dairy cows for the measurement of immune biomarkers. CVM samples were collected from the vagina using gloved hand during the first two weeks postpartum and processed with buffer alone or buffer containing different concentrations of the reducing agents recommended in standard protocols: Dithiothriotol (DTT) or N-Acetyl-L-Cysteine (NAC). Total protein was measured using the bicinchoninic acid (BCA) assay; interleukin 6 (IL-6), IL-8 and α1-acid glycoprotein (AGP) were measured by ELISA.

**Results:**

We found that use of reducing agents to liquefy CVM affects protein yield and the accuracy of biomarker detection. Our improved protocol results in lower protein yields but improved detection of cytokines and chemokines. Using our modified method to measure AGP in CVM we found raised levels of AGP at seven days postpartum in CVM from cows that went on to develop endometritis.

**Conclusion:**

We conclude that processing CVM without reducing agents improves detection of biomarkers that reflect uterine health in cattle. We propose that measurement of AGP in CVM during the first week postpartum may identify cows at risk of developing clinical endometritis.

## Background

The postpartum bovine uterus is susceptible to diverse pathologies including viral and bacterial infection as well as endometritis, all of which impact negatively on the health, productivity and fertility of cows [1–3]. Current diagnostic methods for predicting uterine inflammation such as uterine cytology and biopsy require specialist expertise and invasive tools. In contrast, CVM could provide a useful resource for the analysis of uterine health. CVM is composed of a mixture of oviductal, uterine, cervical and vaginal secretions and their production is influenced by health status, the microbiome and pregnancy [2, 4, 5]. Biomolecules in CVM reflect the health and secretions of the uterus and CVM could therefore substitute for more invasive analyses.

Cytokines, e.g. interleukin 6 (IL-6), chemokines e.g. IL-8 and acute phase proteins e.g. α1-Acid Glycoprotein (AGP), produced by endometrial epithelial cells and local immune populations are increased in inflamed uterine tissue and in vitro models [6–8]. These inflammatory biomarkers have been detected in uterine mucus and CVM [8–10]. Due to the physical properties of mucus, processing of CVM with reducing agents is routinely recommended before the analysis of soluble-phase biomarkers [4, 9, 11]. N-acetyl-L-cysteine (NAC) and Dithiothreitol (DTT) are commonly used to homogenize mucus by reducing the disulfide bonds of mucins [12–14]. However, many immune biomarkers also have disulfide bonds [15, 16] and their detection is likely to be compromised by use of reducing agents. The overall objective of the current study is to improve processing of postpartum CVM for measuring candidate biomarkers that may predict uterine inflammation and disease.

## Results

### Total protein and SDS-PAGE electrophoresis

**Fig. 1:**
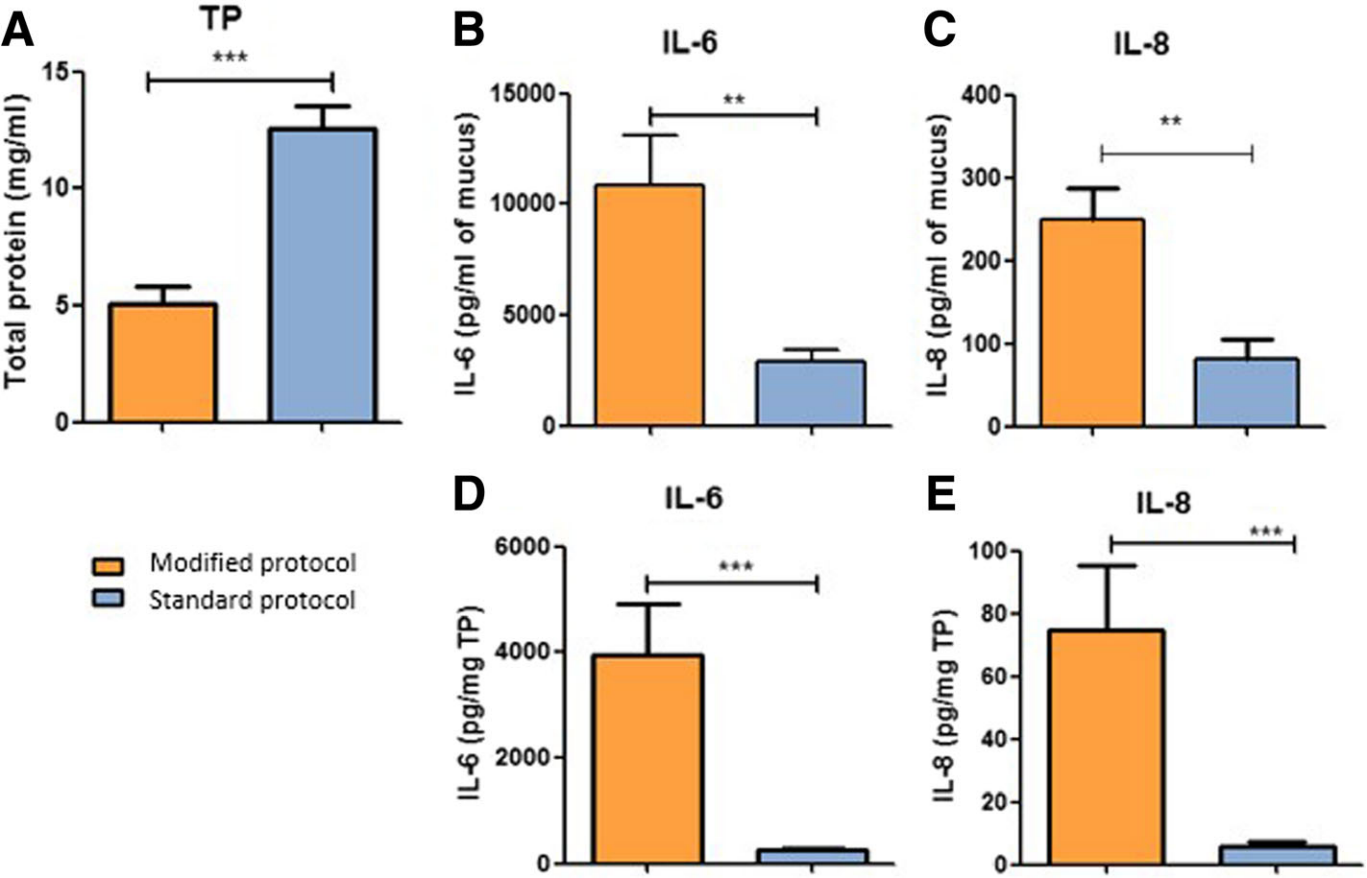
**a** Total protein levels in CVM from 16 cows at first two weeks postpartum were measured using BCA assay and compared between our modified protocol and the standard protocol (with DTT). **b**, **c** IL-6 and IL-8 levels were measured by ELISA and results were presented according to the volume of mucus analyzed. **d**, **e** taking in consideration the amount of total protein (TP) in CVM, IL-6 and IL-8 levels were presented per mg of total protein in the mucus. Results are presented as mean ± SEM and analyzed by *t* test. Significant differences between groups are calculated. ***P < 0.001, **P < 0.01 and *P < 0.05

The standard protocol (with DTT) resulted in higher levels of total protein than the modified protocol (Fig. 1a).

**Fig. 2:**
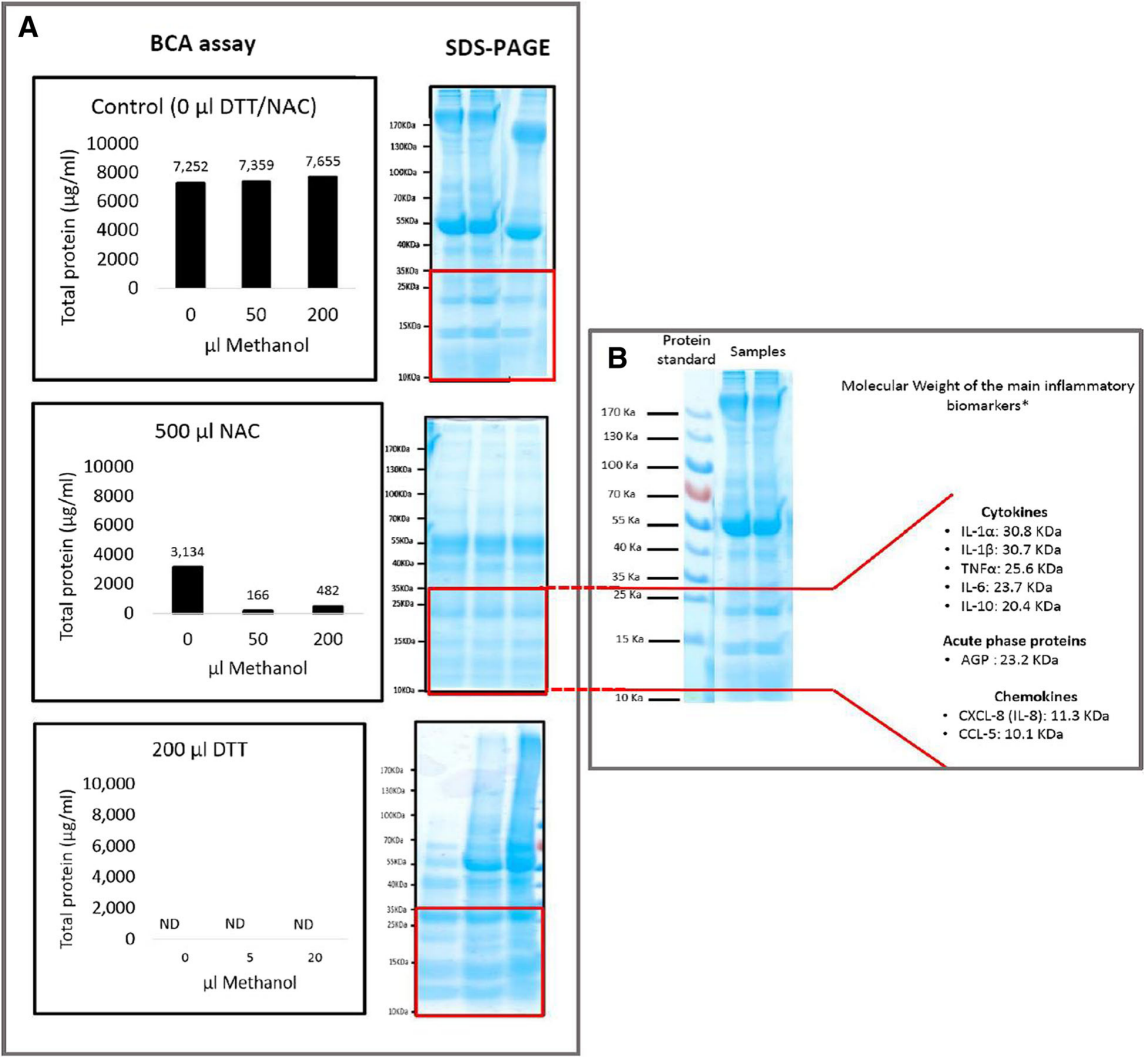
**a** CVM from 16 cows was processed by different protocols, from the top: modified protocol without reducing agent, standard protocol with NAC and standard protocol with DTT. Total protein was analyzed by SDS-PAGE using 4–20% gradient running gel and non-reducing loading buffer to illustrate the protein bands within the range of main inflammatory biomarkers (red box). Different amount of methanol was added. Each protein column from the SDS-PAGE gel corresponds to a bar in the BCA assay graphs. **b** Molecular weight of main inflammatory biomarkers implicated in uterine inflammation; interleukin 1-α, IL-1β, Tumor necrosis factor alpha (TNFα), IL-6, α-1-acid glycoprotein (AGP), IL-10, chemokine (C-X-C motif) ligand 8 (CXCL-8/IL-8) and Chemokine (C-C motif) ligand 5 (CCL-5). ND: not defined. KDa; kilodalton.*www.uniprot.org

To determine which protocol best preserves the integrity of proteins with molecular weights of the candidate cytokines, protocols were compared based on the protein pattern within the range of 10–35 KDa (Fig. 2b) and results are shown in Fig. 2a (red box). Furthermore, processing mucus without reducing agent gave denser band patterns for proteins with large molecular weight (> 130 KDa).

### Measurement IL6, IL8 and AGP

Our modified protocol resulted in the detection of significantly higher levels of both IL-6 and IL-8 in postpartum CVM per ml of mucus (*P* < 0.001) (Fig. 1b and c). Accounting for total protein concentration in each sample, the concentration of IL-6 and IL-8 per mg of total protein was also significantly higher in the modified protocol compared to the standard protocol (*P* < 0.001) (Fig. 1d and e).

**Fig. 3:**
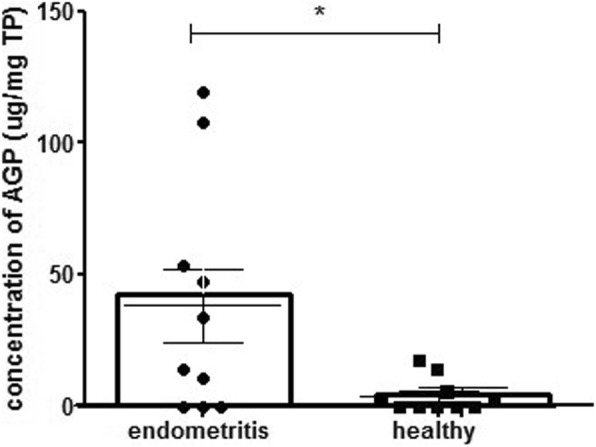
CVM mucus from 20 cows at 7 days postpartum was processed without reducing agent and α1-acide glycoprotein (AGP) levels were measured using ELISA. AGP level was presented according to the amount of total protein (TP) in CVM. Results are presented as mean ± SEM and analyzed by *t* test. Significant differences between groups are calculated. *P < 0.05

AGP levels in CVM processed without reducing agent were higher in cows which went on to develop clinical endometritis compared to healthy cows (*P* < 0.05) (Fig. 3).

## Discussion

Here we demonstrated that use of reducing agents during the processing of CVM impacts on the accurate detection of proteins and biomarkers. The standard protocol (with DTT) resulted in higher levels of total protein than the modified protocol, since DTT is a powerful reducing agent that homogenizes mucus through the reduction of disulfide bounds. Due to the low level of circulating estrogen, postpartum mucus is characterized by high viscosity [5, 17]. For this reason, we believe that DTT used in standard protocols resulted in higher levels of total protein as it helped to breakdown disulfide bonds of the viscous mucins and made protein accessible for measurement by BCA assay. Thus, if for any reason the use of reducing agent is required, it is important to determine the lowest concentration that does not interfere with the marker being assessed.

SDS-PAGE confirmed that use of reducing agent affected the stability of proteins in CVM. CVM samples were run using non-reducing loading buffer to limit interaction with the effect of the reducing agent used for processing mucus. Within the molecular weight range of the main inflammatory cytokines, the protein bands were denser in the modified protocol indicating that they contain higher levels of proteins at this molecular weight. SDS-PAGE shown that for high molecular weight proteins, such as acute phase proteins and mucins, both reducing agents should be avoided as these biomarkers would be reduced and degraded and could not be accurately measured by ELISA.

Our modified protocol resulted in the detection of significantly higher levels of both IL-6 and IL-8 in postpartum CVM. As proteins, cytokines, chemokines and acute phase proteins contain disulfide bonds between the cysteine residues, and the use of reducing agent may affect the stability of these proteins and decrease their detection using ELISA technique. For example, the IL-6 protein contains 4 cysteine residues, which are conserved between different species (i.e. human and cow) and are connected by 2 disulfide bonds (Cys 44-Cys 50 and Cys 73-Cys 83) [18, 19]. The two disulfide bridges can be reduced and alkylated under chemical reduction [20] or non-denaturing conditions [21]. Therefore, reducing agents decrease the stability of IL-6 and may decrease its detection by antibodies, since the disulfide bonds seem to be responsible for maintaining structural integrity of receptor binding sites rather than conformational stability [20, 21]. In a previous study at high concentrations, DTT decreased by 43% the detectable concentration of IL-6 standard [15]. Likewise, IL-8 contains 4 cysteines that form 2 disulfide bonds and it is rapidly inactivated when their disulfide bonds are reduced [22] which would decrease its detection by ELISA.

Increased AGP levels are associated with uterine infection and have been proposed to be prognostic of endometritis [23, 24]. To validate our improved protocol to identify pathological inflammation, AGP was measured in CVM collected at seven days postpartum from 20 cows, 10 of which went on to develop endometritis. CVM processed with the modified protocol resulted in higher levels of AGP in cows which went on to develop clinical endometritis, compared to healthy cows. AGP has two disulfide bounds between cysteine (Cys) Cys5-Cys165 and Cys72-Cys147 [25] and use of reducing agent would breakdown these bounds and decrease the detection of AGP. Likewise, DTT was confirmed to reduce the molecular weight of pig AGP when it used for 2D electrophoresis [26]. Using our improved protocol to measure AGP in CVM to predict cows at risk of developing clinical endometritis, we found that the test could have a sensitivity of 70% and specificity of 100%. Thus, all cows positively identified at day 7 developed clinical endometritis at 21 DPP. These findings could help to reduce antibiotic use by reducing the numbers of cattle treated using CVM diagnosis alone, as well as reducing the costs associated with reproductive diseases.

## Conclusion

Here we show that processing CVM without any reducing agent allows for more accurate measurement of inflammatory biomarkers in early postpartum mucus. We also show that raised AGP levels predict cows at risk of developing clinical endometritis. Thus, the improved detection of biomarkers in CVM from the postpartum cow represents a technique with significant practical utility for early disease diagnosis in a non-invasive and welfare friendly manner.

## Methods

### Herd identification

Material was obtained from 36 mixed-parity Holstein-Friesian dairy cows during their first two weeks postpartum. Animals used in this study belong to three commercial dairy farms. Among these cows, 16 belong to one farm were used to measure IL-6 and IL-8, and 20 cows from two different farms were diagnosed for clinical endometritis by scoring mucus aspect and odor at day 21 postpartum [24] and used to measure AGP levels. All animals were examined in their normal farming conditions and they remain on farm after the samples were collected.

### Vaginal mucus collection

Vaginal examination and mucus collection were performed according to a previously described protocol [27]. Briefly, using examination sleeves, the perineum was wiped and washed with 70% ethanol to remove fecal material. An examination sleeve was covered with clean surgical glove and the gloved hand was inserted through the vulva into the vagina and CVM was collected and scored as described by Williams et al. (2005). CVM was collected in an empty sterile 20 ml tube and immediately placed on ice and transported to the laboratory within 4-6 h.

### CVM processing

CVM collected from 16 cows from one farm was used to optimize the technique and processed using two different protocols: the standard protocol using reducing agent (DTT) [9, 15] and our modified protocol without reducing agent. In the modified protocol, CVM was centrifuged at 3000 x g for 15 min at 4 °C and 500 μl of the upper part was collected and mixed with 1000 μl of sterile PBS in 2 ml eppendorf tubes and vortexed. In the standard protocol (with DTT), CVM was processed according to Cronin et al. (2010). Briefly, 2 g of frozen CVM was added to 10 ml of cytolyt solution (40% methanol: 60% distilled water) and mixed with 1 mM DTT to disrupt the mucus. Tubes were then centrifuged at 3000 x g for 15 min at 4 °C. The supernatant was collected and multiple aliquots of 1 ml were prepared and used to measure IL-6, IL-8 and AGP by ELISA.

CVM from one cow was selected for further analysis using a second reducing agent NAC [15]. In the modified protocol, 500 mg from frozen CVM were mixed with 1000 μl of PBS. In the standard protocol, 500 mg of frozen CVM were mixed with 1000 μl PBS in 5 ml tubes and different volumes of 1 mM NAC (50, 100, 200, 300, 500 μl) or DTT (20, 50, 100, 200 μl) were added to each tube. Tubes were centrifuged at 3000 x g at 4 °C for 15 min and the supernatant was collected and aliquoted into Eppendorf tubes and stored at − 80 °C for further analysis by BCA assay for total protein and SDS-PAGE.

### Measurement of total protein

Total protein levels in CVM were measured using a bicinchoninic acid (BCA) assay using a commercially available kit (Pierce™ BCA Protein Assay Kit (#23227, ThermoScientific®, 3747 N. Meridian Rd. Rockford, IL 61101, United States) according to manufacturer’s guidelines. Briefly, the contents of one albumin standard (BSA) ampule was diluted with PBS into eight standards and one blank. The working reagent was prepared by mixing 50 parts of BCA reagent A with 1 part of BCA reagent B (50:1, Reagent A: B). Then, 25 μL of each standard or sample were added in duplicate into a microplate well. 200 μL of the working reagent was added to each well and the 96 well plate was mixed thoroughly on a plate shaker for 30 s. The plate was then covered and incubated at 37 °C for 30 min. After cooling the plate, the absorbance was measured at 562 nm on a plate reader (GloMax®-Multi Detection System, Promega Corporation, 2800 Woods Hollow Road Madison, WI 53711 USA).

### SDS-PAGE electrophoresis

To visualize the range of proteins in mucus, a gradient running gel 4–20% was chosen in combination with a 5% stacking gel. To not interact with the effect of the reducing agent added to the mucus, samples were loaded in the gel cassette using non-reducing loading buffer. Gels were run at 110 V for 90 min and then stained with Coomassie blue G250 for one hour with gentle agitation. After overnight distaining in 10% glacial acetic acid, gels were scanned and interpreted.

### Measurement IL6, IL8 and AGP

After thawing, samples were centrifuged at 3000 x g for 15 min at 4 °C and the supernatant was used to measure biomarkers. Levels of IL-6, IL-8 and AGP in CVM were measured using commercial ELISA kits (human IL-8 ELISA kit: R&D Systems Inc., Minneapolis, Minnesota, USA; bovine IL-6 ELISA kit: #ESS0029, ThermoScientific®, 3747 N. Meridian Rd. Rockford, IL 61101; Cow AGP: #AGP-11, Life Diagnostics Inc.®, P.O. Box 5205, West Chester, Pa. 19380) according to the guidelines provided by the manufacturers and modified according to previous studies in bovine [6, 9, 10]. Human IL-8 ELISA was used because no bovine specific IL-8 ready-to-use ELISA is commercially available. Furthermore, the antibodies used in the kit have been confirmed to cross-react with bovine IL-8 [6, 28]. To validate the usefulness of the improved protocol to detect uterine health problems, AGP was measured in CVM from 20 animals of which 10 developed clinical endometritis.

### Statistical analysis

Statistical analysis was performed using GraphPad® Prism 5 software (GraphPad Software, Inc. 7825 Fay Avenue, Suite 230 La Jolla, CA 92037 USA). A Students *t* test was used to compare results between two groups, while one-way ANOVA with Bonferroni post-comparison test was used to compare between three or more groups. Results were presented as mean ± SEM and considered statistically significant at *P*-value < 0.05.

## Abbreviations

AGP: α1-acid glycoprotein
BCA assay: Bicinchoninic acid assay
CVM: Cervico-vaginal mucus
Cys: Cysteine
DTT: Dithiothriotol
ELISA: Enzyme-linked immunosorbent assay
IL: Interleukin
NAC: N-Acetyl-L-Cysteine
SDS-PAGE: Sodium dodecyl sulfate polyacrylamide gel electrophoresis
SEM: Standard error of the mean
